# Using the Kalman Algorithm to Correct Data Errors of a 24-Bit Visible Spectrometer

**DOI:** 10.3390/s17122939

**Published:** 2017-12-18

**Authors:** Son Pham, Anh Dinh

**Affiliations:** Department of Electrical and Computer Engineering, University of Saskatchewan, Saskatoon, SK S7N 5A9, Canada; anh.dinh@usask.ca

**Keywords:** spectrometer, spectrum, Kalman, filter, noise reduction, corrector

## Abstract

To reduce cost, increase resolution, and reduce errors due to changing light intensity of the VIS SPEC, a new technique is proposed which applies the Kalman algorithm along with a simple hardware setup and implementation. In real time, the SPEC automatically corrects spectral data errors resulting from an unstable light source by adding a photodiode sensor to monitor the changes in light source intensity. The Kalman algorithm is applied on the data to correct the errors. The light intensity instability is one of the sources of error considered in this work. The change in light intensity is due to the remaining lifetime, working time and physical mechanism of the halogen lamp, and/or battery and regulator stability. Coefficients and parameters for the processing are determined from MATLAB simulations based on two real types of datasets, which are mono-changing and multi-changing datasets, collected from the prototype SPEC. From the saved datasets, and based on the Kalman algorithm and other computer algorithms such as divide-and-conquer algorithm and greedy technique, the simulation program implements the search for process noise covariance, the correction function and its correction coefficients. These components, which will be implemented in the processor of the SPEC, Kalman algorithm and the light-source-monitoring sensor are essential to build the Kalman corrector. Through experimental results, the corrector can reduce the total error in the spectra on the order of 10 times; for certain typical local spectral data, it can reduce the error by up to 60 times. The experimental results prove that accuracy of the SPEC increases considerably by using the proposed Kalman corrector in the case of changes in light source intensity. The proposed Kalman technique can be applied to other applications to correct the errors due to slow changes in certain system components.

## 1. Introduction

The VIS SPEC applies Beer-Lambert law to find the substance concentration [1]. Sensitivity and accuracy of a SPEC depend on several factors, one of these is, firstly, the analog-to-digital converter (ADC) which is responsible for digitalizing the measured analog signal from the sensors and providing resolution to the spectrometer [2]. Secondly, another important factor that affects the accuracy are the system stability factors such as light intensity, voltage regulator, ambient and operating temperature. The stability of the system can be improved by smoothing techniques and data correction methodology [3,4].

Most of SPECs on the market use a 16-bit ADC and a charge-coupled device and CMOS sensors, which account for their high prices [4,5]. Therefore, to increase the SPEC’s sensitivity and to reduce the cost, an ADS1252 [6] and BPW-34 [7] are chosen.

Many approaches and techniques have been used to increase quality of the SPEC [3]. The change in the light intensity emitted from the 12V-50W halogen lamp [8,9] and drift of the lead acid battery [10], which is used to make the SPEC portable, are the causes of error in such devices. Though, the light intensity from the lamp in the device is controlled by a voltage regulator to keep it stable, the emitted light intensity may still fluctuate for some reasons such as the usable-remaining lifetime, working time, and physical mechanism of the halogen lamp [8,9] and the battery. In addition, ambient temperature changes can directly affect the light emission from the halogen lamp [9] and the battery capacity [10] which can also possibly influence the light intensity from the light source. All these factors can cause the light intensity to be unstable at some ranges as the measurement time goes on. If this instability happens during a spectral measurement, the intensity of the investigated spectrum will be unstable as well, and the recorded digital data will contain errors which correspond to the light intensity change. 

We note that the errors in the data, which have the causes previously mentioned, are different from the errors caused by photon shot noise, dark noise, and Johnson or thermal noise [11,12] in which the contribution of shot and Johnson noises dominate the dark noise. For an exposure period, which is smaller than five minutes, the dark noise and dark current are minor and negligible [13]. The Johnson noise can be tackled by applying transimpedance amplifiers [3,12], whereas the shot noise can be treated by low-pass filter circuits [3] or digital filters such as the moving-average filter [14]. Since these noises in a SPEC were previously discussed in [3] and in other references [11,12], in this work, the noises or also-called errors of data possibly caused by the light intensity instability and quantization are investigated. 

Through experimental observation, two main types of light intensity change which were seen during experiments are the mono-trend style, which can be the result of the gradual weakening of the battery, or the physical-working mechanism of the halogen, and the multi-trend style which can be caused by the waving-output voltage for the lamp and is not as quick as a shot noise. Although the output voltage is regulated by a voltage regulator, small or uncommonly-large amplitude oscillations can be seen, that may result from unideal conditions such as unsteady temperature, unstable load, or poor-quality capacitors [15]. As the halogen lamp is 12 V–50 W and the operating voltage is around 12 V, the average current that goes through the lamp is around 4 A. Moreover, the lamp must be warmed up at least 15 min to 30 min before use [16,17]. In addition, after a 15-min warm-up, the output voltage of the battery, which is full charge before use, starts dropping [12]. In the next period of 33 min, the voltage drop will be approximately to 1V, i.e., about 30 mV/min. 

Consequently, the instability can affect to the quality of the spectral measurement without our awareness. As a result, it is essential to monitor the light source intensity to compensate for the change in intensity in order to reduce errors in a SPEC. In this design of the SPEC, a second photodiode is inserted in the light path to monitor its potential light intensity changes. By observing the light intensity change, spectral data and its errors caused by the light changes, one can recognize the correlation between them. Thus, by studying such correlation, the spectral error can be corrected or minimized. This study is implemented by using MATLAB simulations, in which the program uses the Kalman algorithm and the proposed correction functions to find necessary parameters such as process noise covariance. The parameters and coefficients are to be used in hardware implementation of the SPEC. 

## 2. Methodology

**Figure 1:**
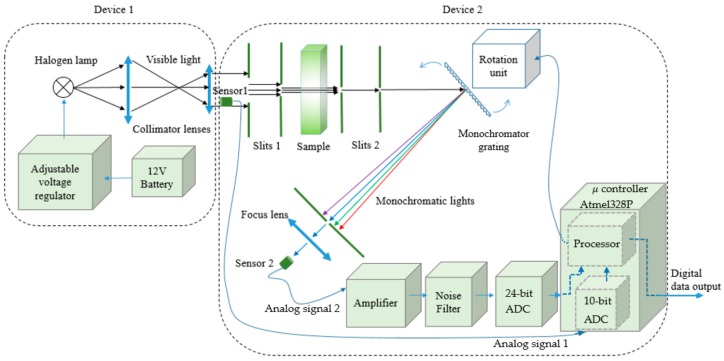
Block diagram of the VIS SPEC. Device 1 provides visible light and device 2 analyzes sample spectrum and sends digital data to a computer.

**Figure 2:**
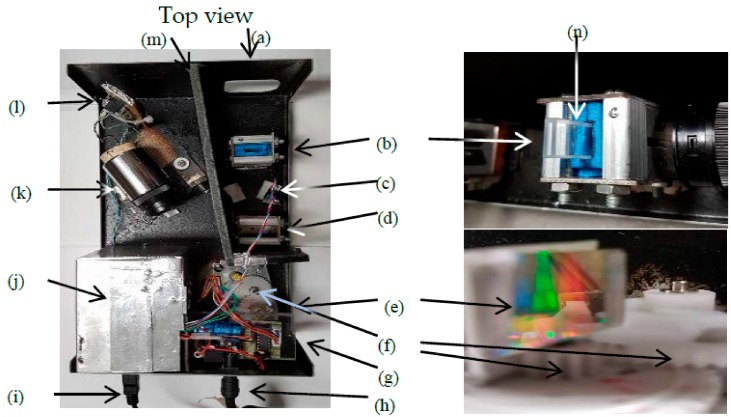
Device 2 components: (**a**) light entrance; (**b**,**d**) slits on the two sides of each unit; (**c**) sensor 1; (**e**) grating and its close view; (**f**) gears are used to increase the scanning steps, one gear is attached to the grating; (**g**) 5 V regulator circuit supply energy for other electronics units inside the device 2, and the driver circuit using ULN2003 IC controls the stepper motor; (**h**) power supply input; (**i**) digital data output; (**j**) metal box protects inner circuits from external noise; (**k**) sensor housing; (**l**) amplifier circuits are combined two low-pass filters which filter out noise greater than 50 Hz; (**m**) wall protects the sensor 2 area from the light entrance area; (**n**) sample cuvette.

In this work, the VIS SPEC structure is shown in the diagram of Figure 1. The system is separated into device 1 and device 2 for convenience in implementation, experiment, and observation. In device 1, the battery supplies energy for the halogen lamp through an adjustable voltage regulator having 3 power 2N1544 transistors [18] to provide load current and the regulator LM317 [19] to control the output voltage. The maximum current through these three transistors is around 15 A and the output voltage can be adjusted from around 6 V to 12 V. In device 1, lens 1 and lens 2 with the focuses of about 61.5 ± 0.5 mm and 36.0 ± 0.5 mm respectively collimate the light from the halogen lamp. Figure 2 shows more details of device 2. 

In device 2, the collimated light from the device 1 goes through the slits units which are designed with metal blades. These slits are used to decrease the cross section of the light into thin-slit light to reduce the diffractive effect [20]. In between the first two slits, there is a sample holder whereby a 1 mm-wide-quartz cuvette is inserted. When the light goes through the sample, the sample absorbs energy of certain wavelengths of the incident light and the remained light provides useful information about the sample. However, to get this information, this transmitted light must be continuously processed by another process where the light shines onto a monochromator, which is a reflective grating, and is separated into mono lights. The period of the grating is 747 ± 11 nm. The grating can be deviated from the incident light by the stepper motor [20] having 4096 steps/round. Moreover, the modified-5V 28BYJ-48 stepper motor [21] having 82,944/round can drive the grating to deviate from the incident light. Thus, each rotating step equals to 75.752 × 10^−6^ radian or 0.0043 degree. Based on the grating, wavelength of the light can be calculated by using the relating formula among incident light, mono diffracted light and the grating period:

(1) $$m\lambda =d\left(\sin\theta_{i}+\sin\theta_{m}\right)$$

where *m* is diffraction order, *d* is grating period, *θ_i_* is incident angle, and *θ_m_* is diffraction angle. 

The mono lights are then directed to sensor 2 which is a photodiode, BPW-34, the diode is used to measure the intensity of the mono lights. The signal from sensor 2 is then properly amplified and filtered by the amplifier and the two low pass filters before being collected, processed and filtered again to eliminate noise by the 24-bit ADC and the Atmel328P microcontroller [22]. From Figure 1, the signals from sensor 1 and sensor 2 after amplified and filtered by the electronic circuits are sent to ADCs and the digital signals then enter the microcontroller. 

Now the details of the error correction mechanism are presented. Basically, the light-source monitoring sensor 1 collects the light-source intensity data, *x*, and the spectral sensor 2 records spectral data, *y*, to send to the microcontroller for further processing. In case, the light-source intensity is stable, there are no errors for both *x* and *y*. Therefore, *x* = $x_{real}$, and *y* = $y_{real}$, where $x_{real}$ and $y_{real}$ are the real value of *x* and *y* respectively. If the light intensity is unstable, then $x\neq x_{real}$, and $y\neq y_{real}$. The difference of these values can be defined as error values, and:

(2) $$dx=x-x_{real};\;dy=y-y_{real}$$

From experiments, when the light intensity is decreased, the spectral intensity is also decreased; and, inversely, when the light intensity is increased, the spectral intensity is also increased. Therefore, *y* is proportional with *x*.

Consequently, from Equation (2), it can be seen that *dy* is proportional with *dx*, so *dy ~ dx*. Thus, *dy* = *f*(*dx*), and:

(3) Y=y+dy=y+f(dx)

where *Y* is the corrected data, and *f*(*dx*) is considered as correction function. *f* can be proportional with dx, |dx|2∗dx|dx|, $dx^{3}$, …, or |dx|1/2∗dx|dx|, $dx^{1/3}$, .... Because the form of *f* is not know yet, let assume:

(4) f(dx)=Co∗dx+C1∗|dx|2∗dx|dx|+C2∗dx3+…+C1′∗|dx|1/2∗dx|dx|+C2′∗dx1/3+…

where, $C_{o}$, $C_{1}$, $C_{2}$,…, $C_{1}^{\prime }$, $C_{2}^{\prime }$, …are proportional constant values which must be estimated by simulation on MATLAB software to have optimal coefficients. dx|dx| has only two values, −1 or +1, and indicates *dx* is negative or positive. To support for a successful simulation, some algorithms and technique are manipulated.

### 2.1. Greedy Technique

The way to find out these constants’ values is simple. It is based on the greedy technique [23] in which Equation (4) is used in the simulation. At first, all $C_{o}$, $C_{1}$, $C_{2}$, …, $C_{1}^{\prime }$, $C_{2}^{\prime }$, … are set to zero. Then, $\Delta C_{o}$, $\Delta C_{1}$, $\Delta C_{2}$,…, $\Delta C_{1}^{\prime }$, $\Delta C_{2}^{\prime }$, … are the increment values of $C_{o}$, $C_{1}$, $C_{2}$, …, $C_{1}^{\prime }$, $C_{2}^{\prime }$, … respectively, and set to certain small values. With the greedy technique, the simulation program will start to find a certain proportional constant. Let’s start with $C_{o}$. The program will add $\Delta C_{o}$ into $C_{o}$, then substitute $C_{o}$ into Equation (4) to calculate *dy* = *f*(*dx*). After that, put *dy* into Equation (3) to have the corrected data, *Y*, and then compare *Y* with $y_{real}$ to see whether *Y* is good or not. Theoretically, *Y* is good when *Y* equals $y_{real}$. However, this is impossible when there are many other factors such as circuit noise can influence to the estimation process. Thus, to know when the proportional estimation process should stop, some criteria such as absolute error (AR), relative error (RE), error (ERR), least square (LS), or correlation parameter (CP) [14] have been applied, where the definitions of AE, RE, ERR, or LS are:

(5) $$\mathrm{AE}=\sum_{i=1}^{N}\;\left|Y\left(i\right)-y_{real}\left(i\right)\right|;\mathrm{RE}=\sum_{i=0}^{N}\left|Y\left(i\right)-y\_real\;\left(i\right)\right|⁄y\_real;\;\mathrm{ERR}=\sum_{i=1}^{N}\left(Y\left(i\right)-y_{real}\left(i\right)\right);\mathrm{LS}=\sum_{i=1}^{N}{\left(Y\left(i\right)-y_{real}\left(i\right)\right)}^{2}$$

where *i* is the index of data vector *Y* and $y_{real}$, and *N* is the number of data points, as *Y* and $y_{real}$ are discrete data. Let’s take AE for instance, after estimate the AE, *a prior* AE is compared with *a posterior* AE. If *a posterior* AE < *a prior* AE, then the estimation of $C_{o}$ still need to be processed again by adding it with $\Delta C_{o}$. Inversely, the program will stop to search for $C_{o}$ and move to another proportion constant. This process keeps going until all $C_{o}$, $C_{1}$, $C_{2}$, …, $C_{1}^{\prime }$, $C_{2}^{\prime }$, … are found to fulfill *f*. 

The advantage of the greedy technique is that it is feasible to apply, but the disadvantage is that the solution, *f*, and its correction coefficients results from the simulation program, are probably not the best ones. For example, with a certain suggested *f*, if the order of searching the correction coefficients for *f* is $C_{o}$, $C_{1}$, $C_{2}$, …, $C_{1}^{\prime }$, $C_{2}^{\prime }$, … respectively, then the result is supposedly $f_{1}$. Again, changing the order of searching into $C_{2}$, $C_{1}$, $C_{o}$, …, $C_{2}^{\prime }$, $C_{1}^{\prime }$, …, one can get $f_{2}\neq f_{1}$. Thus, if *n* orders of searching are conducted, there can be *n* different forms of *f*. To determine which *f* is the better one, the above criteria are looked at. Consequently, among these forms there should be the best one. 

In practice, when the form of the correction function is long and complicated, then it is not efficient to code in a microcontroller. Moreover, it also increases running time in the measurement which may lead to further error. For example, the running time for a measurement of more than five minutes produces dark noise [11] and/or potential light-intensity errors during that time. Therefore, in practice, several cognitive short forms of *f* are proposed for the simulation program. 

### 2.2. Divide and Conquer Algorithm

From experiments, the correction function found from the greedy technique cannot effectively and adequately correct the range of the spectrum of interest. To circumvent this difficulty, the investigated range is divided into smaller subdomains. At each of these subdomains, there will be a correction function and corresponding parameters to conquer it. This technique is called divide-and-conquer algorithm [24]. Therefore, data error of each subdomain will be mitigated by the function. Supposedly, there are *n* subranges, and so, there are *n* correction functions. In the operation program, the main data domain *R* is cut into twelve subdomains, *R_i_*:

(6) $$\{\begin{array}{c} R_{1},\;R_{2},\ldots ,R_{11},R_{12}\leq R\\ R_{1}⋃R_{2}⋃\ldots ⋃R_{11}⋃R_{12}=R \end{array}$$

Theoretically, the smaller the subranges are divided, the better the corrected data are. However, when the number of the subranges increases, the time which is used to process data will be longer. There must be a balance among measuring time, the number of subranges, and the measured data. In this work, through experiments, twelve subranges are formed to perform the data correction task. As mentioned above, to achieve the best results, a MATLAB simulation will be used to search for them. In the MATLAB code, some subrecursive functions are built and recalled continuously in the simulation. In general, the problem is just shrinking into smaller domains to get better simulation results which serve for the later steps. 

### 2.3. Kalman Algorithm

There is one more obstacle still able to hinder the search for the best $C_{o}$, $C_{1}$, $C_{2}$, …, $C_{1}^{\prime }$, $C_{2}^{\prime }$, …. In practice, after running the searching simulation with the raw data *x*, to get *f*, and applying it to correct the spectral data, the results are not as satisfactory as expected, since when the raw light-source-monitoring data, *x*, which could have noises [11,12,13], quantization error [14], and unstable light-intensity error, are sent to *f*, not only is the unstable light-intensity error transformed by *f* but also by the mixture of noises and quantization error. These noises and quantization error will make the operating criteria (AE, RE, ERR, LS, or CP) work ineffectively and inadequately. Consequently, *x* must be treated by certain approaches before use. 

Moreover, the chosen approach should be applicable and feasible for the microcontroller code and satisfies real-time application without any processing lag and procrastination. For feasibility, two outstanding algorithms are the moving average [14] and the Kalman algorithm [23,25,26]. For real-time applications, the Kalman algorithm has been proved by some simple experiments to dominate the moving average algorithm. After being treated by the Kalman algorithm, ideally, *dx* (*dx* = $x-x_{real}$) containing only unstable light-intensity error, which is considered as useful information, will be ready to serve for the correction process. 

Details of the Kalman algorithm can be found in [23,25,26], but it is necessary to focus on some Equations and quantities of the theory for later applying explanation. First, the system is supposed to be linear [26,27], so its state equation has the form:

(7) $$X_{k}=A_{k}X_{k-1}+B_{k}U_{k}+W_{k}$$

where $X_{k}$ and $X_{k-1}$ are the state vector, and $\in R^{n},A_{k}$, is the *n* × *n* state transition matrix, $B_{k}$ is the optional *n* × *l* control input matrix, $U_{k}$ is the control vector, and $\in R^{l}$, and $W_{k}$ is the process noise vector. The observation vector of the system is:

(8) $$Z_{k}=H_{k}X_{k}+V_{k}$$

in which $Z_{k}$ is the observation or measurement vector, and $\in R^{m}$, $H_{k}$ is the *m* × *n* observation matrix, and $V_{k}$ is the measurement noise vector. Then, the noise happening in the device is assumed to be white noise in the frequency domain, while in the time domain, its probability density has the Gaussian shape at each point on the time axis [27]. The normal probability distributions of $W_{k}$ and $V_{k}$ are:

(9) $$\{\begin{array}{c} p\left(W_{k}\right)\sim N\left(0,Q_{k}\right)\\ p\left(V_{k}\right)\sim N\left(0,R_{k}\right) \end{array}$$

where $Q_{k}$ and $R_{k}$ are process-noise covariance and measurement-noise covariance, respectively. Their normal probability density functions have the form of:

(10) $$f\left(x\right)=\frac{1}{\sqrt{2\pi \sigma_{x}}}exp\left[-\frac{x^{2}}{2\sigma_{x}^{2}}\right]$$

where *x* stands for one state of the state vector or one observation of the observation vector. $\sigma_{x}$ is the standard deviation of *x* [28]. From stochastic theory, in a deviation range and a three-time deviation range from the mean, there will contain 68.27 percent, and 99.73 percent of the measured values, respectively [29,30]. Therefore, from the comment and the experiment error theory [31], the error can be approximated to the three-time deviation. From [29,32], the standard deviation of the sample is:

(11) $$\sigma =\sqrt{\frac{\sum_{j}^{N}{\left(x_{j}-\bar{x}\right)}^{2}}{N}}$$

with:

(12) $$\bar{x}=\frac{1}{N}\overset{N}{\underset{j}{\sum }}x_{j}$$

According to [14], *a priori* and *a posteriori* estimate errors can be defined respectively as:

(13) $$e_{k}^{-}=X_{k}-{\hat{X}}_{k}^{-}$$

(14) $$e_{k}^{+}=X_{k}-{\hat{X}}_{k}^{+}$$

where ${\hat{X}}_{k}^{-}\in R^{n}$ is the *a priori* estimate at discrete time ***k*** providing information of the previous process, and ${\hat{X}}_{k}^{+}\in R^{n}$ is the *a posteriori* estimate at discrete time ***k*** providing information of the measurement $Z_{k}$. From (13) and (14), the covariance of a priori error and the covariance of *a posteriori* error are respectively:

(15) $$P_{k}^{-}=E\left[e_{k}^{-}e_{k}^{-T}\right]$$

(16) $$P_{k}^{+}=E\left[e_{k}^{+}e_{k}^{+T}\right]$$

The *a posteriori* estimate has the form [11,12,14] of:

(17) $${\hat{X}}_{k}^{+}={\hat{X}}_{k}^{-}+K_{k}\left(Z_{k}-H_{k}{\hat{X}}_{k}^{-}\right)=\left(1-K_{k}H_{k}\right){\hat{X}}_{k}^{-}+K_{k}Z_{k}$$

where $(Z_{k}-H_{k}{\hat{X}}_{k}^{-}$) is the residual or measurement innovation which shows the difference between the measurement and a priori estimate, and $K_{k}$, named Kalman gain or blending factor, is chosen by minimizing the covariance in (16). From [26,27,32], $K_{k}$ is:

(18) $$K_{k}=P_{k}^{-}H_{k}^{T}{\left(H_{k}P_{k}^{-}H_{k}^{T}+R_{k}\right)}^{-1}$$

**Figure 3:**
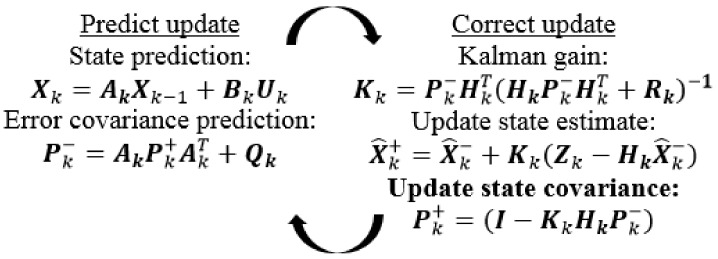
Kalman filter operation loop.

From (18), when the measurement noise covariance is small, $Z_{k}$ has high fidelity and the Kalman gain will be large. At that time, from (17), the weight coefficient of $Z_{k}$ is greater than the weight of ${\hat{X}}_{k}^{-}$, so ${\hat{X}}_{k}^{+}$ will “*believe*” more in $Z_{k}$ than ${\hat{X}}_{k}^{-}$. Inversely, as $P_{k}^{-}$ is small, the Kalman gain will be small and ${\hat{X}}_{k}^{+}$ will “*believe*” more in ${\hat{X}}_{k}^{-}$ than $Z_{k}$. Figure 3 shows the operation loop of the Kalman algorithm.

In practice, $Q_{k}$ and $R_{k}$ can be estimated before applying the Kalman filter. In the study, $R_{k}$ is calculated by several rough measured data, but $Q_{k}$ is determined by MATLAB simulation to get the best results. These values, during operation, can be constant, so they are presented without the discrete-time subscript *k*. From [23,25,26], one can see that $R_{k}$ = $\sigma^{2}$. In practice, $R_{k}$ is easily determined by using Equations (11) and (12). 

### 2.4. Performance Description

In this section, all the processes manipulated to process data with the order of correction function, process noise covariance, and correction coefficients findings, by MATLAB simulation are presented. 

**Figure 4:**
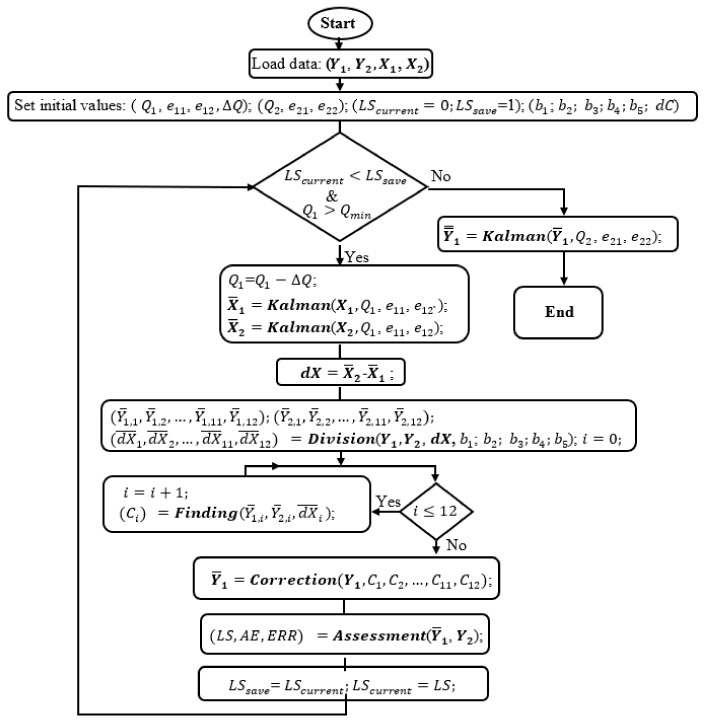
The simulation flowchart to find correction parameters or process noise covariance $Q_{1}$.

First, in Equations (1), (2), and (4), the values of real data of sensor 1 and sensor 2 were mentioned and known. However, that is only a hypothesis to help establish the processing algorithm. In practice, the values measured in stable conditions are considered approximately equal to the real values and are reference values to serve for comparison and calculation in simulation. The flowchart in Figure 4 shows the core algorithm for the simulation program, where $Y_{1}$ and $Y_{2}$ are spectral data measured in unsteady conditions and good conditions respectively, whereas $X_{1}$ and $X_{2}$ are light intensity data recorded in these two cases. Conventionally, $X_{1}$ and $Y_{1}$ are data with unstable-light-intensity data with error, and $X_{2}$ and $Y_{2}$ are reference data. 

Second, noticing that $Y_{1}$ and $Y_{2}$ are 24-bit data, while $X_{1}$ and $X_{2}$ are 10-bit data. Then, $Q_{1}$, $e_{11}$, and ΔQ, respectively are process noise covariance, measurement error, and the decrement of $Q_{1}$ sequentially. Especially, $e_{12}$ takes two roles, *a posterior* error and a priori error in the ***Kalman*** module. Many data, $X_{1}$, $X_{2}$, $Y_{1}$, and $Y_{2}$, were measured and saved in datasets before the simulations. $LS_{current}$ and $LS_{save}$ are the least square parameters which are used to operate the loop. $b_{1}$, $b_{2}$, …, and $b_{5}$ which are the fixed values are set to satisfy condition:

(19) $$0<b_{1}<b_{2}<\ldots <b_{5}<Y_{max}$$

and they are considered as “boundary” values to help divide the grand spectral data domain into subdomains. In the simulation program, the maximum potential number of the subdomains which are non-empty is 12, if the minimum data value is smaller than $b_{1}$, the maximum data value is greater than $b_{5}$, and at any range of (0, $b_{1}$), [$b_{1}$, $b_{2}$), …, [$b_{4}$, $b_{5}$), [$b_{5}$, $Y_{max}]$ both positive and negative ***dX*** values exist. $C_{1}$, $C_{2}$, …, $C_{12}$ are the correction parameters, whereas *dC* is the increment of these parameters.

#### 2.4.1. Correction Function Finding

As discussed above, to make the correction function feasible and applicable, several short forms of Equation (4) are proposed. They are sequentially replaced into the simulation program which is illustrated by the flowchart in Figure 4. Thus, the simulation program will help to find the correction coefficients for the suggested functions. After finishing each simulation, the program will return not merely $Q_{1}$, $C_{1}$, $C_{2}$, …, $C_{12}$, but also AE, ERR, LS, or CP which can be used as the quality assessment criteria of the correction functions. Therefore, which correction function with the prominent assessment parameters is to be adopted. The simulation program starts by loading data $X_{1}$, $X_{2}$, $Y_{1}$, and $Y_{2}$ from the assigned datasets and initiating the values for $Q_{1}$, $e_{11}$, $e_{12}$, ΔQ, $LS_{current}$, $LS_{save}$, $b_{1}$, $b_{2}$, …, $b_{5}$, and *dC*. 

In the flowchart, $X_{1}$ and $X_{2}$ are processed by the ***Kalman*** algorithm to mitigate the quantization error and electronic noises, which may remain, albeit being filtered by hardware filters, to keep the light-source-intensity information as clean as possible. The searching simulation is not successful if the light-source data still have high quantization error and noises. Furthermore, to empower the corrector to work effectively and adequately, the data from sensor 1 and sensor 2 should be collected by applying unstable-light-intensity styles from gradual to fast changes and from mono-changing, to multi-changing styles with large enough fluctuation amplitudes to use in the simulation. However, at this stage, the correction coefficients of the correction function are the priority, so $Q_{1}$ is cognitively set to a certain value that is good enough for the simulation and of course, larger than $Q_{min}$. ΔQ is set to zero to not influence on $Q_{1}$.

**Figure 5:**
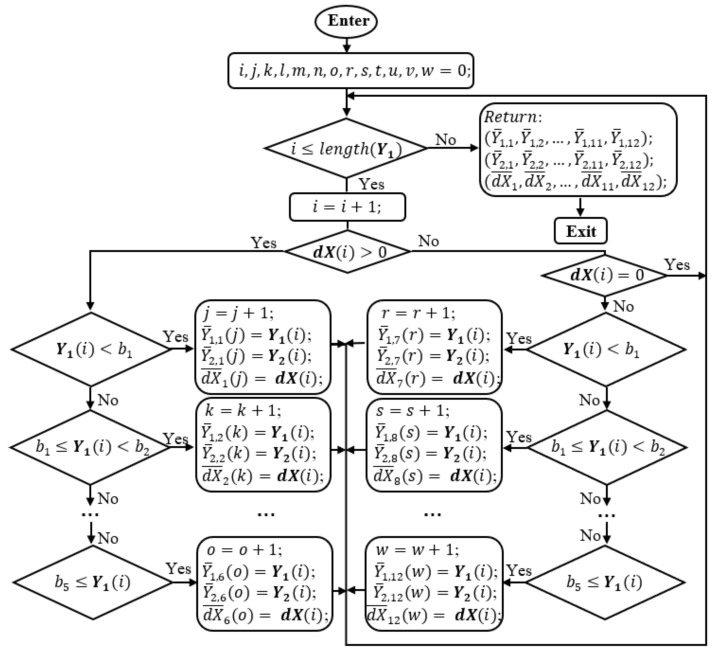
The diagram of the Division function.

Let’s look at the operation in the flowchart of Figure 4. At first, $LS_{current}$ = 0 < $LS_{save}$ = 1 and $Q_{1}$ > $Q_{min}$ satisfy the main condition in the flowchart. Then, $Q_{1}$ is decreased by a ΔQ = 0, so it does not change. $X_{1}$ and $X_{2}$ are smoothed by the ***Kalman*** module that returns ${\bar{X}}_{1}$ and ${\bar{X}}_{2}$ respectively prior to calculate ***dX*** = ${\bar{X}}_{1}-{\bar{X}}_{2}$. Then, the ***Division*** function is called to divide dX, $Y_{1}$, and $Y_{2}$ into smaller domains. At this point, the divide-and-conquer algorithm is applied to separate the grand data domain into smaller domains which are more easily to conquer and to find the solution. The way to divide the data is illustrated in the flowchart of Figure 5.

In this function, there are thirteen indexes, *i*, *j*, *k*, *l*, *m*, *n*, *o*, *r*, *s*, *t*, *u*, *v*, *w* to address data points in the vector dX, $Y_{1}$, and $Y_{2}$. Initially, *i* is smaller than the number of the data elements of $Y_{1}$ that is checked by the first condition in Figure 5. *i* is increased one unit and the condition of whether dX(*i*) is positive is checked. With either “Yes” or “No”, this first data element, $Y_{1}\left(1\right)$ is compared with $b_{1}$, $b_{2}$, $b_{3}$, $b_{4}$, and $b_{5}$ to see to which data subdomain it must belong to. The difference here is that if dX(*i*) > 0, the first six subdomains, ${\bar{Y}}_{1,1}$, ${\bar{Y}}_{1,2}$, …, ${\bar{Y}}_{1,6}$ for $Y_{1}$, ${\bar{Y}}_{2,1}$, ${\bar{Y}}_{2,2}$, …, ${\bar{Y}}_{2,6}$ for $Y_{2},\;{\bar{dX}}_{1,1}$, ${\bar{dX}}_{1,2}$, …, ${\bar{dX}}_{1,6}$ of dX, are used for the arrangement. In case dX(*i*) < 0, the second six subdomains, ${\bar{Y}}_{1,7}$, ${\bar{Y}}_{1,8}$, …, ${\bar{Y}}_{1,12}$ for $Y_{1}$, ${\bar{Y}}_{2,7}$, ${\bar{Y}}_{2,8}$, …, ${\bar{Y}}_{2,12}$ for $Y_{2},\;{\bar{dX}}_{1,7}$, ${\bar{dX}}_{1,8}$, …, ${\bar{dX}}_{1,12}$ of dX, are used for the assembly. When dX(*i*) = 0, the running point will jump back to the first condition. Then, the same procedure is repeated until *i*, loop index, is larger than the number of the data elements of $Y_{1}$. Especially, when all vector dX = 0, the division will return twelve empty subdomains. In this case, there is no need to fix the spectral data. When all dX > 0, from Figure 5, the left side condition boxes will be conducted. Consequently, the first six subdomains are none-empty, and the second six subdomains are empty. Inversely, when all dX < 0, the second six subdomains are none-empty, and the first six subdomains are empty.

The data subdomain of order *i*, ${\bar{Y}}_{1,i},\;{\bar{Y}}_{2,i},\;{\bar{dX}}_{i}$, are loaded. In this process, $ls_{curent}$ and $ls_{save}$ are also the least square values to serve for operating the loop. When $ls_{curent}$ < $ls_{save}$ and ${\bar{Y}}_{1,i}$ is different with null, $C_{i}$ is increased by a *dC*. $ls_{curent}$ < $ls_{save}$ condition means the current corrected sub data is better the previous corrected data, so this sub data still can be better amended. Then, the suggested correction function is applied to fix ${\bar{Y}}_{1,i}$ and returns ${\overset{=}{Y}}_{1,i}$. Next, the subprogram will call the ***Assessment*** function to calculate LS, AE, ERR based on Equation (5), the corrected data, ${\overset{=}{Y}}_{1,i}$, and reference data, ${\bar{Y}}_{2,i}$, and updates $ls_{save}$ with $ls_{curent}$, and $ls_{curent}$ with LS. Then, the subprogram keeps going back to the operating condition until $ls_{curent}$ > $ls_{save}$ which means the current corrected data cannot be further corrected. From the flowchart in Figure 4, the finding module are called twelve times to access all the subdomains without noting of whether they are null or not.

After escaping from the finding module and having all correction coefficients, the main simulation grogram will apply these values to correct all the sub data, ${\bar{Y}}_{1,1}$, ${\bar{Y}}_{1,2}$, …, ${\bar{Y}}_{1,6}$, of the grand spectral data, $Y_{1}$ by calling the ***Correction*** function which is built from the suggested correction function, *f*. The Equation is in the form:

(20) $${\overset{=}{Y}}_{1,i}\left(j\right)={\bar{Y}}_{1,i}+C_{i}*f[({\bar{dX}}_{i}\left(j\right),{\bar{Y}}_{1,i}\left(j\right)]$$

Then, the ***Assessment*** function will help to evaluate LS, ERR, AE, or CP by using $Y_{1}$ and $Y_{2}$. Thus, it is similar to the above description of how the least square criterion works that the program will not stop when $LS_{current}$ < $LS_{save}$. 

Finally, at the end, the simulation will return many coefficients, but the most interesting coefficients are LS, ERR, AR, and CP. Therefore, with each suggested short-form correction function, they are the most interesting coefficients. Assessing these coefficients, the best one is chosen to be used in the correction function.

#### 2.4.2. Process Noise Covariance Finding

After finding the appropriate correction function from the suggested functions, the simulation program is applied again to find the process noise covariance, $Q_{1}$. Although, $Q_{1}$ used above is good enough, it may not be the best to assure the light-source-intensity data as clean as possible. As mentioned above, many types of data will be used to serve the simulation. Currently, ΔQ is cognitively set to a certain value which is small enough for the searching simulation. The procedure exactly repeats what is described in Section 2.4.1, except for the main operating condition must account for the condition $Q_{1}<Q_{min}$, and at every loop, $Q_{1}$ is decreased by a ΔQ. When the condition $LS_{current}$ < $LS_{save}$ and $Q_{1}<Q_{min}$ cannot be satisfied, the simulation program will cease to return $Q_{1}$. This value of process noise covariance is to be expected that can keep the best light-source data from the noise. 

#### 2.4.3. Correction Coefficients Finding

After the above crucial steps, the main simulation program, which having the flowchart depicted in Figure 4, simply reinstalls the found correction function and $Q_{1}$ back into itself to find $C_{1}$, $C_{2}$, …, $C_{12}$. Before that, ΔQ is set to zero. At the end, it returns the correction coefficients.

#### 2.4.4. Application

**Figure 6:**
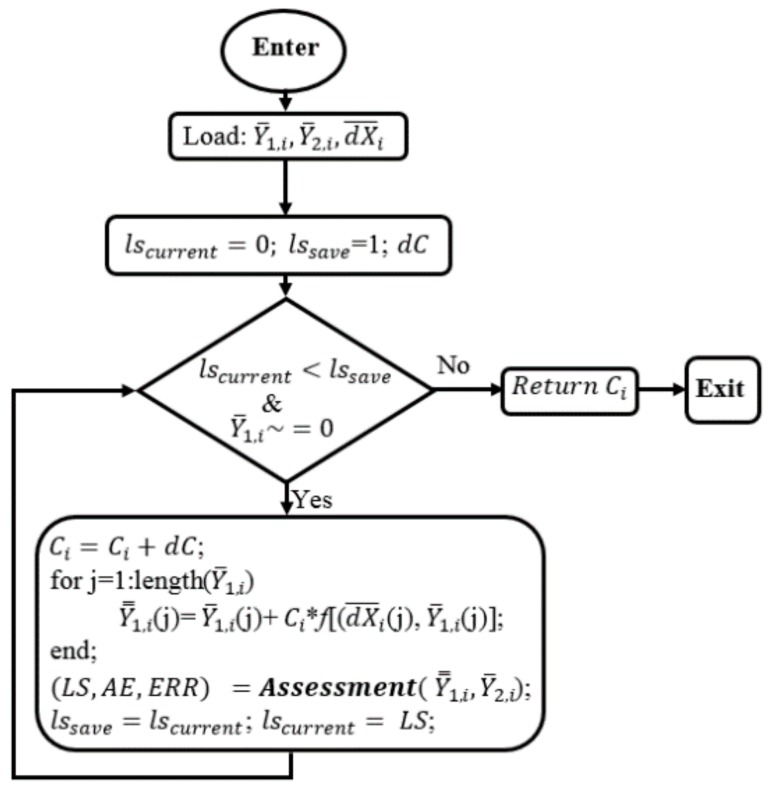
The *Finding* flowchart for correction parameters.

**Figure 7:**
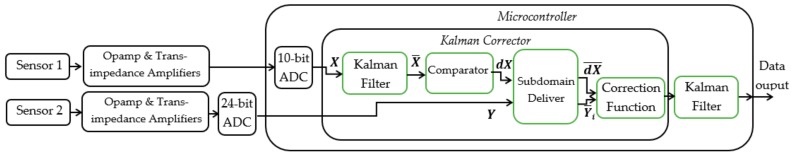
The main roles of the Kalman algorithm and their correlation with other parts.

After the simulation step, all necessary key components, correction function, correction coefficients, and process noise covariance are provided and ready to apply in the device and to build the Kalman corrector. Figure 7 illustrates the procedure of collecting and processing data. The light intensity signal from sensor 1 enters the 10-bit ADC and the spectral intensity signal from sensor 2 goes to the 24-bit ADC. These signals are digitalized to become digital data. ***X*** is filtered out noises and quantization error by $Q_{1}$ to become $\bar{X}$. Next, the compare block will calculate ***dX*** which $dX=X_{o}-X$, where $X_{o}$ is a loosely optional value. $X_{o}$ can be equal to a certain standard value or a measured value which is measured ahead before any spectral measurement. Then, ***dX*** and ***Y*** values will be delivered by the subdomain deliver to assigned data subdomains which are characterized by $b_{1}$, $b_{2}$, …, $b_{5}$ and controlled by ***dX***. Here, the subdomain deliver works similar to the division. It will base on whether ***dX*** is positive or negative and to what range among (0, $b_{1}$), [$b_{1}$, $b_{2}$), …, [$b_{4}$, $b_{5}$), [$b_{5}$, $Y_{max}]$ ***Y*** belongs to. ***Y*** is then sent to the correction function which is governing the data subdomain corresponding with that range (Section 2.4.1 and Figure 6). For example, if ***dX*** > 0, and 0 < ***Y*** < $b_{1}$, then ***Y*** is sent to the corresponding subdomain, where is governed by a data correction function of $Y_{1}$ founded by the previous simulation, to be thoroughly amended. This is performed in real time, so ***dX*** and ***Y*** are no longer data vector but rather discrete-time data measured at each discrete time.

## 3. Results

The light source in this study is visible, so its spectrum ranges from 450 nm to 750 nm. For convenience in the following figures, on the vertical axis, the unit for the light intensity or spectra is an arbitrary unit (a.u). With the spectral intensity plot, the horizontal axis presents the step unit corresponding to the scanning steps of the stepper motor instead of wavelength unit or number wavelength unit which can be changed among them by using Equation (1). The step value is changed to rotation angle and the angle is translated into wavelength. However, in the light-source intensity plots, the step value on the horizontal axis simply corresponds to the discrete time values in the sampling signal.

As described earlier, the light intensity may increase or decrease randomly when the voltage regulator is not working well or changes due to other reasons such as physical conditions, ambient air temperature, lamp temperature, or warm up and working time. The changes can also display a mono trend, caused by some conditions such as the drift of the battery. Before running the simulation program, many experimental data have been recorded to serve the simulations. The recorded data are collected under unchanging and slightly changing working conditions to simulate the unstable factors as mentioned in the Introduction which can lead to slight changes in light intensity. Then data recorded in good working conditions (external stable power supply) serve as reference data for the assessment process. 

### 3.1. Initial $Q_{1}$ Selection for Simulation

**Figure 8:**
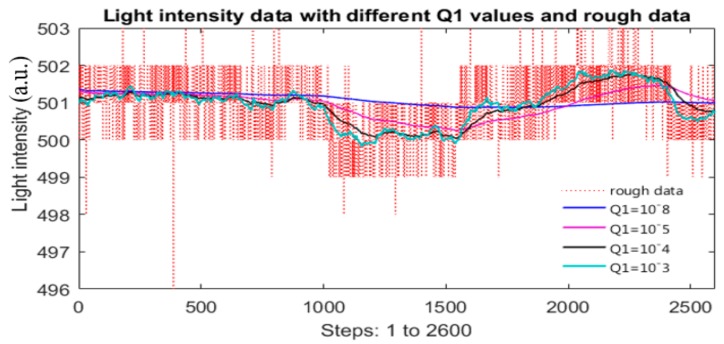
Raw intensity data and its filtered data with different $Q_{1}$ values.

In Figure 8, the red-dot line is the recorded raw 10-bit data of the light intensity source which was slightly adjusted to simulate an unstable light source. Several values of $Q_{1}$ are tested to study the features of $Q_{1}$. One may see that the smaller the $Q_{1}$ is, the smoother output data from the Kalman function will be. This leads to that $X_{1}$, the blue line, is so smooth, when $Q_{1}$ is so small, for example $Q_{1}=10^{-8}$ (it is matching with this type of data and measurement error). Thus, not only the electronics noises and quantization error are removed but also some useful and crucial information from the light source. Obviously, this will ruin the data correction and simulation process.

When $Q_{1}=10^{-3}$, the filtered data, ${\bar{X}}_{1}$, plot is the cyan line. Currently, ${\bar{X}}_{1}$ keeps much light-intensity information, but also some electronic noises and quantization error as well. If using this filtered data for simulation, the results, such as correction coefficients, or corrected spectral data, may not be the best. 

**Figure 9:**
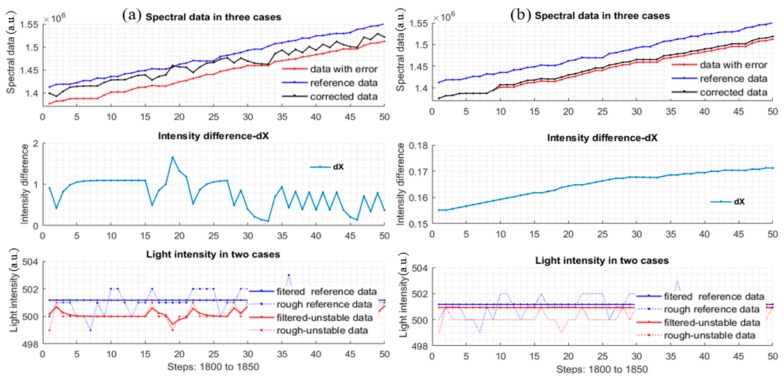
Results of measurement data and processed data (**a**) the three plots of data in the case of ***Q*_1_** = 0.9; and (**b**) the three plots of data in case of ***Q*_1_** ≈ 1.27 × $10^{-21}$.

Therefore, neither too large nor too small $Q_{1}$ can lead to good results which are demonstrated by plot groups of Figure 9a,b, respectively. In both cases, the corrected data are not close to or similar to the reference. Section 2.4.2 shows how to obtain an appropriate and adequate $Q_{1}$. To serve for the simulation of finding correction function which will be discussed in the next section, $Q_{1}$ is set to 10^−3^.

### 3.2. Correction Function Choice

A multi-changing *data set 1* is loaded for test. The general initial values are: *b* = 7 × 10^5^, *b*_1_ = *b*, *b*_2_ = 2 × *b*, *b*_3_ = 3 × *b*, *b*_4_ = 3.6 × *b*, *b*_5_ = 4 × *b*, $Q_{1}$ = 10^−3^, $e_{11}=e_{12}=1$, and $e_{21}=e_{22}=500$ (experimentally determined by Equations (11) and (12)). With each correction function which is chosen, the increment, *dC*, of the correction parameters must be cognitively adjusted to a certain appropriate value that assures that the finding function will be called at least several times. This value should not be so small because the simulation process may last too long without getting better results. In the next section, four typical correction functions are investigated to select the most appropriate one in these. These proposed functions are based on experiment, evaluation, and observation of the results to adjust logically. Objectively, these correction functions are tested with the same *dataset 1*. Notice that, LS, AE, ERR, and CP are the controlling criteria that are also used as effective and qualitative parameters for the decision.

In Table 1 below, $f_{A}$, $f_{B}$, $f_{C}$, and $f_{D}$ are short correction function forms. The formulas of these functions are given in Appendix. The **LS**, **AE**, and **ERR** are expected as small as possible, i.e., the corrected spectral data are similar to the reference spectral data. For **CP**, if the two data signals are very close to each other, **CP** will be approximately one. In the table, an auxiliary index is **Feasibility**. Simply, it takes the relative evaluation values which are based on the form of the correction function.

If **Feasibility** is high, the function is easily applied. In some cases, this index can strengthen a decision, albeit merely an auxiliary one. In the table, the prominent values are highlighted. Observing Table 1, one sees the most outstanding correction function is possibly $f_{D}$, and the second one is $f_{B}$, finally, $f_{D}$ is selected. 

### 3.3. Process Noise Covariance Search

After the correction function, ${\overset{=}{Y}}_{1,i}$(*j*) = ${\bar{Y}}_{1,i}$(*j*) + $C_{i}$ ∗ ${\bar{dX}}_{i}$(*j*) ∗ ${\bar{Y}}_{1,i}\left(j\right)$, has been chosen, the process noise covariance, $Q_{1}$, search is the next step. As previously discussed, to achieve an appropriate and adequate of $Q_{1}$, many datasets which were measured under different light-intensity-changing styles are used. The multi-changing *dataset 2* to *dataset 19* (named by the authors) are chosen to be loaded into the simulation program. Table 2 provides the values of $Q_{1}$ gathered after the simulations. 

From the above $Q_{1}$ search simulations corresponding with multi-changing data sets, a $Q_{1}$ will be chosen. There will be some points of view in choosing $Q_{1}$. For instance, one may suppose that the average $Q_{1}$ partly satisfies all the cases of light intensity change, or the greatest $Q_{1}$ of the found values is the safe solution as the Kalman filter can catch up the possibly fastest and, obviously, slowest intensity change of the studied data sets. However, with the later philosophy, $Q_{1,max}$ will be greater than the average one, ${\bar{Q}}_{1}$, and then the corrected data pertaining to $Q_{1,max}$ is probably not as smooth as the corrected data of ${\bar{Q}}_{1}$. If $Q_{1,min}$ is selected, there will probably be some quick-changing-light intensity not detected well by the Kalman filter. To implement in the device, the selected process noise covariance is ${\bar{Q}}_{1}$ = 0.0203. After selecting the best correction function and the appropriate $Q_{1}$ for the Kalman module, the next process is to load the mono-changing data sets to find the correction parameters, *C_i_*.

### 3.4. Correction Parameters Finding

In this section, the mono-changing datasets are considered. In these datasets, there are two subtype data where one has spectral and light intensity data greater than the reference spectral and light intensity data, and the other one has spectral and light intensity data smaller than the reference spectral and light intensity data. Thus, with these types of dataset, after division module is called, there should be six continuous empty data subdomains and six continuous non-empty data subdomains. The upper-subdomain data and lower-subdomain data are investigated separately to find the correction parameters. The values of *C_i_* of the upper-subdomain and lower-domain data are collected by simulation and shown in Table 3 and Table 4, respectively.

Notice that *dX* is adjusted from around the range of −40 to 30 units corresponding to the potential change of the light intensity. Each unit corresponds to approximately a 4.9 mV difference when the 10-bit ADC of the microcontroller is used. For a small change in light intensity, the relationship of *dX* and *C_i_* is expected to be linear with that the best applied correction function, ${\overset{=}{Y}}_{1,i}$(*j*) = ${\bar{Y}}_{1,i}$(*j*) + $C_{i}$ ∗ ${\bar{dX}}_{i}$(*j*) ∗ ${\bar{Y}}_{1,i}\left(j\right)$. 

**Figure 10:**
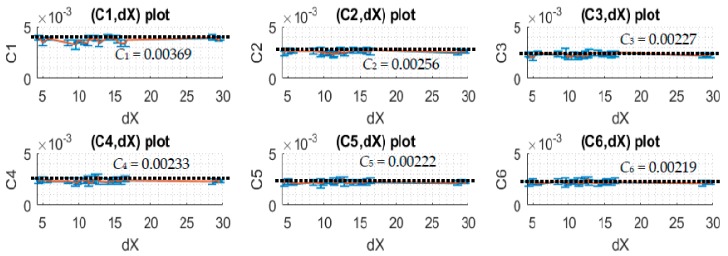
The plots of correction coefficients and *dX* of upper-subdomain data.

**Figure 11:**
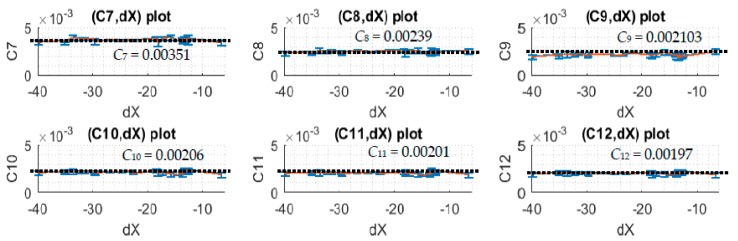
The plots of the correction coefficients and *dX* of lower-subdomain data.

When *dX* is adjusted to different positive and negative values which correspond to the simulations of different voltage increases and decreases, one can see from the plots of Figure 10 and Figure 11 that *C_i_* and *dX* relationship is linear as expected and the slope angle is zero. In this case, one can take the mean values of *C_i_* to make it as the correction coefficients of the corresponding subdomains.

### 3.5. Practice

Through experiments and simulations, the correction function below is used:

$${\overset{=}{Y}}_{1,i}\left(j\right)={\bar{Y}}_{1,i}\left(j\right)+C_{i}*{\bar{dX}}_{i}\left(j\right)*{\bar{Y}}_{1,i}\left(j\right),$$

and the noise process covariance, ${\bar{Q}}_{1}$ = 0.0203, and the correction coefficients, *C*_1_ = 0.003709, *C*_2_ = 0.002577, *C*_3_ = 0.002278, *C*_4_ = 0.002368, *C*_5_ = 0.002242, *C*_6_ = 0.002215, *C*_7_ = 0.003694, *C*_8_ = 0.002449, *C*_9_ = 0.002103, *C*_10_ = 0.002155, *C*_11_ = 0.002084, *C*_12_ = 0.002036 are found through the simulation described in the previous sections. 

#### 3.5.1. Dataset Correction Simulation

Now with the all the parameters and function found, one can check to see how the output data from the corrector is better than the uncorrected data. For the process assessment, two new quantities are added:

(21) $$\left|dY_{1}\left|=\right|Y_{2}-Y_{1}\right|$$

(22) $$\left|dY_{2}\left|=\right|Y_{2}-\bar{Y_{1}}\right|$$

Equations (21) and (22) are the absolute errors of the uncorrected data and corrected data, respectively. 

**Figure 12:**
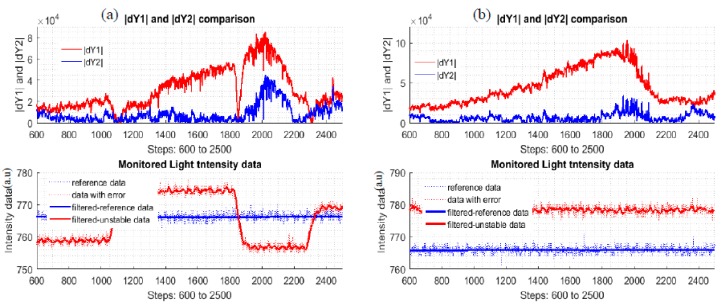
(**a**): $\left|dY_{1}\right|$ and $\left|dY_{2}\right|$ plots of multi-changing *dataset 24*; (**b**): $\left|dY_{1}\right|$ and $\left|dY_{2}\right|$ plots of lower-mono-changing *dataset 1*.

Figure 12 shows the plots of the new quantities, $\left|dY_{1}\right|$, $\left|dY_{2}\right|$ of the multi-changing data and mono-changing data of the two different styles datasets. The expectation here is that the absolute error plots is as close to the zero line as possible. From the plots, the corrected data is better than the uncorrected data. The intensity plots only illustrate how the light intensity was changed to simulate possible reality situations.

#### 3.5.2. Measurements on Air, H_2_O, and KMnO_4_ Samples

**Figure 13:**
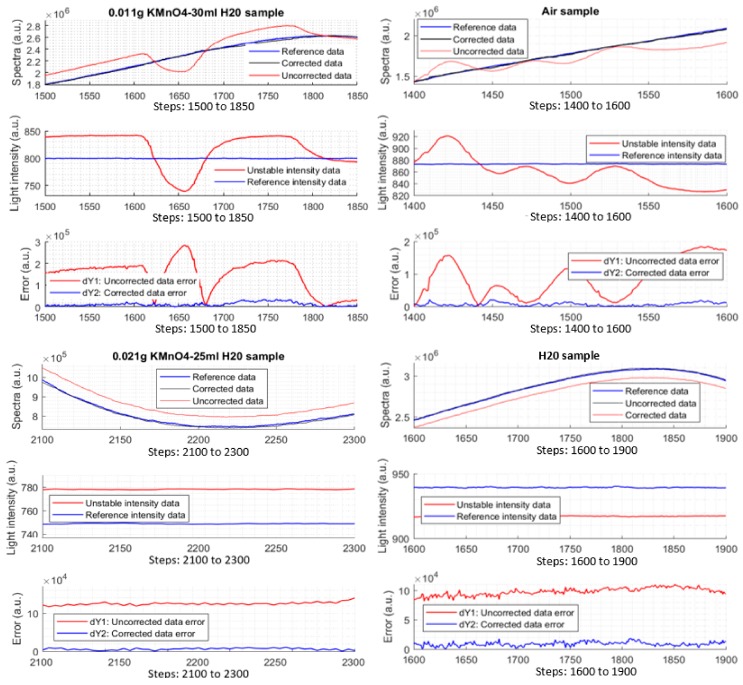
Experimental data of different samples. (**a**) 0.011 g KMnO_4_ and 30 mL distilled H_2_O; (**b**) air; (**c**) 0.021 g KMnO_4_ and 25 mL distilled H_2_O; (**d**) distilled H_2_O.

After the simulation testing, the correction function and the parameters are applied into the device for further testing. For the test, the reference data are still required. The light intensity of the light source is adjusted similarly when the data with error were recorded to serve for the above simulations. The difference here is the data with error will be corrected immediately by the corrector module which is coded into the Atmel328P processor. To check the effectiveness and ability of the corrector, several different types of samples are required (air, H_2_O, and KMnO_4_ samples) under the light intensity changes in both mono-changing and multi-changing strategies. From experiment data, spectral data, light intensity data, and errors are plotted, Experimental results prove that the corrector module works well as can be seen in Figure 13. Note that there are four types of different samples and three types of plots in the figure to illustrate the work of the corrector. The errors of the data after the correction are also plotted. 

By defining the simple formula, *r* = $\frac{\left|dY_{1}\right|\;}{\left|dY_{2}\right|}$, indicates how many times the corrector reduces the light-intensity error. According to calculation from the measured data, *r* ≈ 10 times. At some local data points, it could reach to 60 to 70 times. This coefficient is a used as a merit to evaluate the effectiveness of the Kalman corrector. However, it just shows much meaningful when there is much light-intensity error happening, albeit a good coefficient.

## 4. Discussion and Conclusions

In general, the Kalman algorithm is modified as a corrector to compensate for the data error caused by the unstable light source in a VIS SPEC. The single-beam-24-bit visible spectrometer, which is empowered by an auxiliary photodiode sensor, an appropriate correction function, and Kalman algorithm, can automatically correct the error caused by the light-source-intensity instability which happens randomly or by drifting of the sources. In average, when there is error in the spectral data, it can be reduced approximately by 10 times. The results show a good performance both in simulations and experiments.

One drawback of the technique is the use of the average of $Q_{1,min}$ to $Q_{1,max}$ from the simulation. Section 3.3 provides a range of the applicable values of the noise process covariance. For being able to adapt to the diversity of light-intensity instability, the mean value of the range is selected. Consequently, the data with error may not be perfectly corrected due to either the lack of or not enough light-intensity error information and/or quantization errors still remaining in the light-intensity data. 

To improve the ability of the corrector module, other methods can be applied along with this technique to achieve better results. For example, one can use moveable boundaries, instead of applying the currently fixed boundaries. With moveable boundaries, LS, AE, ERR, and correlation coefficient are still the operation criteria. 

## Figures and Tables

**Table 1:** LS, AE, ERR, and CP of $f_{A}$, $f_{B}$, $f_{C}$, and $f_{D}$ are shown, respectively.

	LS	AE	ERR	CP($Y_{1}$, $Y_{2}$)	CP(${\bar{Y}}_{1}$, $Y_{2}$)	Feasibility
$f_{A}$	2.159 × 10^11^	1.483 × $10^{7}$	2.773 × 10^6^	0.999608	0.999968	Medium
$f_{B}$	1.846 × 10^11^	1.3747 × $10^{7}$	3.3367 × 10^6^	0.999608	0.9999769	Low
$f_{C}$	1.9372 × 10^11^	2.3045 × $10^{7}$	3.3367 × 10^6^	0.999608	0.9999725	Low
$f_{D}$	1.840 × 10^11^	1.3719 × $10^{7}$	2.8859 × 10^6^	0.999608	0.9999756	High

**Table 2:** $Q_{1}$ values of multi-changing datasets.

	*dataset 2*	*dataset 3*	*dataset 4*	*dataset 5*	*dataset 6*	*dataset 7*	*dataset 8*	*dataset 9*	*dataset 10*
$Q_{1}$	0.0071	0.0321	0.0561	0.0147	0.0112	0.0076	0.0352	0.0119	0.0155
	*dataset 11*	*dataset 12*	*dataset 13*	*dataset 14*	*dataset 15*	*dataset 16*	*dataset 17*	*dataset 18*	*dataset 19*
$Q_{1}$	0.0144	0.0349	0.0124	0.0203	0.0126	0.0249	0.0107	0.0165	0.0268

**Table 3:** *C_i_* values of upper-subdomain data.

	$C_{j}$ of Subdomains	$C_{1}$	$C_{2}$	$C_{3}$	$C_{4}$	$C_{5}$	$C_{6}$	$\bar{dX}$
*Data Set*								
1		0.00386	0.00264	0.00245	0.00233	0.00208	0.00207	5.7078
2		0.00345	0.00261	0.00202	0.00246	0.00231	0.00230	4.9686
3		0.00392	0.00240	0.00231	0.00231	0.00206	0.00206	4.4369
4		0.00377	0.00283	0.00204	0.00243	0.00240	0.00227	4.6495
5		0.00356	0.00238	0.00201	0.00248	0.00229	0.00226	10.3858
6		0.00359	0.00242	0.00196	0.00213	0.00206	0.00202	9.8095
7		0.00364	0.0026	0.00227	0.00230	0.00222	0.00216	9.8672
8		0.00328	0.00256	0.00247	0.00222	0.00212	0.00218	9.5766
9		0.00345	0.00264	0.00236	0.00233	0.00220	0.00230	8.5105
10		0.00391	0.00267	0.00238	0.00248	0.00229	0.00231	15.2758
11		0.00393	0.00272	0.00234	0.00225	0.00216	0.00213	14.6995
12		0.00380	0.00277	0.00238	0.00230	0.00224	0.00217	14.5844
13		0.00398	0.00272	0.00247	0.00224	0.00215	0.00210	14.0529
14		0.00391	0.00265	0.00215	0.00257	0.00238	0.00233	12.1743
15		0.00340	0.00249	0.00246	0.00232	0.00215	0.00219	12.7739
16		0.00341	0.00260	0.00236	0.00255	0.00236	0.00236	16.4325
17		0.00341	0.00265	0.00233	0.00234	0.00224	0.00219	15.8561
18		0.00357	0.00259	0.00242	0.00230	0.00215	0.00212	15.3245
19		0.00371	0.00274	0.00252	0.00240	0.00227	0.00224	16.5607
20		0.00342	0.00228	0.00210	0.00249	0.00234	0.00231	11.2676
21		0.00349	0.00248	0.00235	0.00232	0.00228	0.00222	10.7491
22		0.00384	0.00247	0.00234	0.00224	0.00221	0.00216	11.3957
23		0.00368	0.00238	0.00216	0.00243	0.00236	0.00232	12.4826
24		0.00391	0.00233	0.00223	0.00207	0.00208	0.00201	11.3750
25		0.00370	0.00242	0.00212	0.00214	0.00217	0.00211	11.9064
26		0.00386	0.00257	0.00218	0.00231	0.00221	0.00215	29.6674
27		0.00396	0.00254	0.00222	0.00229	0.00216	0.00211	28.5594
28		0.00385	0.00256	0.00221	0.00243	0.00228	0.00226	29.0910
29		0.00366	0.00255	0.00232	0.00222	0.00214	0.00212	29.4339
30		0.00388	0.00264	0.00229	0.00238	0.00224	0.00222	29.1488
**Average**		**0.00369**	**0.00256**	**0.00227**	**0.00233**	**0.00222**	**0.00219**	

**Table 4:** *Ci* values of lower-subdomain data.

	$C_{j}$ of Subdomains	$C_{7}$	$C_{8}$	$C_{9}$	$C_{10}$	$C_{11}$	$C_{12}$	$\bar{dX}$
*Data Set*								
1		0.00355	0.00231	0.00219	0.00187	0.0019	0.00188	−20.2399
2		0.00355	0.00226	0.00182	0.00225	0.00218	0.00209	−13.4950
3		0.00343	0.00253	0.00248	0.00189	0.00189	0.00187	−6.5176
4		0.00336	0.00232	0.00211	0.00209	0.00209	0.00196	−18.0569
5		0.00373	0.00242	0.00211	0.00206	0.002	0.00196	−13.2514
6		0.00381	0.00241	0.00203	0.00207	0.002	0.00197	−13.7458
7		0.00307	0.00226	0.00213	0.00194	0.00193	0.00188	−13.3111
8		0.00307	0.00236	0.00228	0.00203	0.00205	0.00202	−12.9464
9		0.00323	0.00199	0.00171	0.00186	0.00181	0.00172	−10.6810
10		0.00358	0.00251	0.00204	0.00234	0.00223	0.00213	−13.0744
11		0.00309	0.00239	0.00205	0.00197	0.00195	0.00201	−17.5519
12		0.00351	0.00229	0.00215	0.00183	0.00186	0.00183	−13.5530
13		0.00306	0.0021	0.00161	0.00195	0.00189	0.00185	−11.2250
14		0.00368	0.0023	0.00218	0.00185	0.00183	0.00187	−14.6510
15		0.00335	0.00238	0.00223	0.00187	0.00184	0.00188	−13.2710
16		0.00389	0.00253	0.00215	0.00205	0.00198	0.00196	−17.3292
17		0.00374	0.00249	0.00207	0.00227	0.00216	0.00209	−23.2423
18		0.00364	0.00248	0.00209	0.00219	0.0021	0.00201	−24.7748
19		0.00373	0.0026	0.00222	0.00214	0.00208	0.00205	−26.7884
20		0.00364	0.00255	0.00233	0.002	0.00199	0.00197	−22.6055
21		0.00347	0.00232	0.00204	0.00217	0.00212	0.00208	−35.0176
22		0.00341	0.00253	0.00227	0.00205	0.00202	0.00203	−22.3267
23		0.00382	0.00246	0.00207	0.00217	0.0021	0.00202	−32.2190
24		0.00373	0.00243	0.00202	0.00217	0.00209	0.00202	−41.6049
25		0.00347	0.00231	0.00191	0.00210	0.00201	0.00194	−39.7620
26		0.00379	0.00242	0.00198	0.00215	0.00206	0.00199	−29.6439
27		0.00309	0.00242	0.0022	0.00204	0.00197	0.00196	−22.4463
28		0.00361	0.00242	0.00214	0.00217	0.00213	0.00208	−35.0595
29		0.00383	0.00254	0.00227	0.00219	0.00208	0.00208	−31.3988
30		0.00347	0.00239	0.00215	0.0021	0.00197	0.00198	−29.5559
**Average**		**0.00351**	**0.00239**	**0.00210**	**0.00206**	**0.00201**	**0.00197**	

## Appendix A

The short correction forms of the formulas used in Section 3.2, Table 1:

$$f_{A}={\bar{Y}}_{1,i}\left(j\right)+C_{i}*{\bar{dX}}_{i}\left(j\right)*{\bar{Y}}_{1,i}^{2}(j),$$

$$f_{B}={\bar{Y}}_{1,i}\left(j\right)+C_{i}*{\bar{dX}}_{i}\left(j\right)*\sqrt{{\bar{Y}}_{1,i}\left(j\right)},$$

fC=Y¯1,i(j)+Ci∗dX¯i(j)∗Y¯1,i32(j),

$$f_{D}={\bar{Y}}_{1,i}\left(j\right)+C_{i}*{\bar{dX}}_{i}\left(j\right)*{\bar{Y}}_{1,i}\left(j\right).$$

Noticing that the form of $f_{A}$, $f_{B}$, $f_{C}$, and $f_{D}$ is slightly different from Equation (4). As in the experiments, authors found that the correction function work much more effectively if ${\bar{Y}}_{1,i}^{n}\left(j\right)$ is inserted, where *n* is 1, 2, 3, …, or 1/2, 1/3, 3/2. Thus, there is a relation between the correction function *f* with not only ***dX*** but also ***Y***.

## References

[1] Parnis J.M., Oldham K.B. (2103). Beyond the Beer-Lambert Law: The Dependence of Absorbance on Time in Photochemistry. J. Photochem. Photobio. A: Chem..

[2] Microwaves and RF. http://www.webcitation.org/6vjkuW0CJ.

[3] Pham S., Dinh A. Using Trans-impedance Amplifier and Smoothing Techniques to Improve Signal-to-Noise Ratio in a 24-bit Single Beam Visible Spectrometer. Proceedings of the 105th IIER International Conference.

[4] Mightex Co. Miniature CCD Spectrometers with High Resolution and High Stability. http://www.mightexsystems.com/images/File/Mightex_HRS_spectrometer_specifications_Sept2013.pdf.

[5] StellarNet Inc. Analytical Instrumentation—Surf the New Wave in Portable Fiber Optic Spectrometry. http://www.webcitation.org/6vjlqnZpm.

[6] Texas Instrument Inc. Manual: 24-bit, 40 khz Analog-to-Digital Converter. http://www.ti.com/lit/ds/symlink/ads1252.pdf.

[7] Osram Silicon Pin Photodiode Version 1.4. BPW 34 S. http://www.osram-os.com/Graphics/XPic5/00215430_0.pdf/BPW%2034%20S.pdf.

[8] Jaffé H.H., Orchin M. (1962). Theory and Applications of Ultraviolet Spectroscopy.

[9] Tungsten Halogen Lamps. https://www.intl-lighttech.com/specialty-light-sources/tungsten-halogen-lamps-gas-filled-lamps.

[10] PS-1270 12 Volt 7.0 AH: Rechargeable Sealed Lead Acid Battery. http://www.powersonic.com/images/powersonic/sla_batteries/ps_psg_series/12volt/PS1270.pdf.

[11] Fullerton S., Bennett K., Toda E., Takahshi T. Orca-Flash4.0 Changing the Game. Hama. Coper. http://www.webcitation.org/6v3pafaWg.

[12] Osi Optoelectronics Co. Manual: Photodiode Characteristics and Applications. http://www.osioptoelectronics.com/application-notes/an-photodiode-parameters-characteristics.pdf.

[13] What Is Dark Noise?. http://camera.hamamatsu.com/jp/en/technical_guides/dark_noise/index.html.

[14] Smith S.W. (1999). The Scientist and Engineer’s Guide to Digital Signal Processing.

[15] Texas Instrument Inc. Manual: LP2980,LP2982,LP2985-Engineers Note: Capacitors are Key to Voltage Regulator Design. http://www.ti.com/lit/wp/snoa842/snoa842.pdf.

[16] Vo-Dinh T., Gauglitz G. (2003). Handbook of Spectroscopy.

[17] Belton C. Lab Manual: UV-VIS. http://www.webcitation.org/6v70Gyhg7.

[18] 2N1544 Germanium PNP Transistor. http://www.semicon-data.com/transistor/tc/2n/2N1544.html.

[19] LM317 3-Terminal Adjustable Regulator. http://www.ti.com/lit/ds/symlink/lm317.pdf.

[20] Halliday D., Resnick R., Walker J. (1997). Fundamentals of Physics.

[21] 28BYJ-48 Stepper Motor 5VDC. http://www.webcitation.org/6ve05B41c.

[22] Atmel 8-Bit Microcontroller with 4/8/16/32kbytes In-System Programmable Flash. http://www.atmel.com/images/Atmel-8271-8-bit-AVR-Microcontroller-ATmega48A-48PA-88A-88PA-168A-168PA-328-328P_datasheet_Complete.pdf.

[23] Levitin A. (2012). Introduction to the Design & Analysis of Algorithms.

[24] Alur R., Radhakrishna A. Scaling Enumerative Program Synthesis via Divide and Conquer. Proceedings of the Tools and Algorithms for the Construction and Analysis of Systems: 23rd International Conference, TACAS, ETAPS.

[25] Melsa J.L., Cohn D.L. (1978). Decision and Estimation Theory.

[26] Grewal M.S., Andrews A.P. (2008). Kalman Filtering: Theory and Practice Using MATLAB.

[27] Maybeck P.S., George M.S. (1982). Stochastic Models, Estimation, and Control.

[28] Pandit S.M., Wu S.M. (1983). Time Series and System Analysis, with Applications.

[29] Vincent J.D. (1990). Fundamentals of Infrared Detector Operation and Testing.

[30] Harvey A. (1990). Forecasting, Structural Time Series Models and the Kalman Filter.

[31] Carlson G.A. Experimental Errors and Uncertainty, 2002. http://www.webcitation.org/6u7N8FcKs.

[32] Welch G., Bishop G. (2006). An Introduction to the Kalman Filter.

